# The Rate of Addiction in Parents of Toddlers With Congenital Heart Disease

**DOI:** 10.5812/ijhrba.7312

**Published:** 2013-03-12

**Authors:** Iraj Sahramian, Noor Mohammad Noori, Abdolvahab Moradi, Forugh Forghani, Elham Sharafi

**Affiliations:** 1 Department of Pediatrics, Children and Adolescent Health Research Center, Zabol University of Medical Sciences, Zabol, IR Iran; 2 Department of Pediatrics, Children And Adolescent Health Research Center, Zahedan University of Medical Sciences, Zahedan, IR Iran; 3 Department of Microbiology, Gorgan University of Medical Sciences, Gorgan, IR Iran; 4 Department of Gynecology, Zabol University of Medical Sciences, Zabol, IR Iran; 5 Department of Ophtalmology, Zahedan University of Medical Sciences, Zahedan, IR Iran

**Keywords:** Opium, Pregnancy, Heart

## Abstract

**Background:**

Opium abuse is one of the widespread social problems, and one of the most worrying aspects of it is the effect of parents’ drug abuse on the fetus.

**Objectives:**

The present study has investigated the correlation between opium abuse during pregnancy in mothers with congenital heart defects in their children.

**Materials and Methods:**

From early 2009 to late 2011, for two consecutive years in specialized pediatric center of Zahedan Medical University, 225 of children suffering from congenital heart defects were examined and compared with 480 healthy ones for mother opium dependency. The final data were analyzed by student t-test and χ^2^ in SPSS software and the two groups were compared in terms of their parents’ addiction to opium.

**Results:**

From 225 children under study 23.5% had addicted parents based on the variables of the study but the rate was only 2.3 for the control group. The difference between these two groups was significant and the most common form of heart disease was congenital ventricular septal defect.

**Conclusions:**

Opium has teratogenic effect on cardiovascular system.

## 1. Background

Man has known and been using opium since the beginning of human history (1). Ancient civilization epigraphs illustrate the use of poppy in the past and the term opium is derived from the Greek word “theraichos” meaning a mixture of opium and other materials (1). During the recent years, the basic pharmacology of these materials has been identified and besides 20 alkaloids other artificial materials like heroin and methadone and etc. have been produced by man (2). Today, drug abuse is one of the widespread social problems and one of the most worrying aspects of it is the effect of parents’ drug abuse on the fetus (3). Results of some studies suggest that a large number of addicted mothers have had some experiences of drug abuse during pregnancy and also a higher number of women abusing drugs are at pregnancy age (4). These facts are really shocking and drug abuse during pregnancy causes a lot of problems such as congenital malformations, premature babies, still births, meconium aspiration, Neonatal withdrawal syndrome, preeclampsia, Third trimester bleeding, and malpresentation for the fetus and the mothers (5).

Up to now, the conducted studies indicate obvious effects of drug abuse, including opium, on the brain (6). In this process, both reproduction and migration of cells in the beginning of pregnancy and development and growth of the brain in the second phase of pregnancy are greatly influenced (7). It has been found that this negative impact, in addition to the direct poisoning effect of drugs can result from hypoxia hypotension or other side effects of drug abuse (8). Teratogenitic effect of opium on the brain can bring about the possibility of negative effects on other systems and organs like heart and vessels (7) heart and brain’s similar embryonic origin in cephalic region of the embryo and splitting of endocardial cushions cells in the neural crest cells indicates the likelihood of heart’s primary cells being affected by opium (9). Widespread drug abuse in Sistan and Blauchestan region because of its adjacency to Afghanistan, the largest producer of opium in the world (1), lack of information on the cause of many congenital heart problems and scarcity of studies performed on teratogenicity of opium, demands for research in this area.

## 2. Objectives

The present study aimed to investigate the correlation between mothers’ opium abuse during pregnancy with congenital heart defects in their children.

## 3. Materials and Methods

From early 2009 to late 2011, for two consecutive years from in and out-patients in specialized pediatric center of Zahedan Medical University, some children suffering from congenital heart diseases without any other anomalies or diseases were selected. Also among these children the ones whose mothers had no disease during pregnancy or did not use any specific drug , visited their doctors frequently, and had followed necessary hygienic recommendations were selected. After being selected for the study, the above mentioned children had to fill out a questionnaire containing all demographic information and also data on the opium addiction of their parents during pregnancy.

In the current study, opium addition mattered only when the mother was addicted or both parents, father’s addiction was not a criterion. Then, for every child suffering from congenital heart disease qualified to enter the study, two healthy children of the same age who had applied for regular check-ups were selected and their questionnaires were filled out too. After collecting the data form questionnaires, software was employed to analyze the final data by student t-test and χ^2^ the two groups were compared in terms of their parents’ addiction to opium.

## 4. Results

From 225 of patients with heart disease 138 (61.3%) were male and in the control group from 480 cases 293 were male. The average age (month), average height, and average weight are shown in Table 1. In height average with student t - test and P = 0.001, significant difference is noticed (1).

**Table 1.** Average Height, Average Age and Average Weight of Patients and Control Group

Features	Control	Case	P value
**Height average, cm**	92 ± 0.24	86.1 ± 23	0.001
**Weight average, kg**	13.24 ± 10.74	12.41 ± 9.45	NS
**Age average, month**	44.58 ± 43.66	45.36 ± 44.29	-

This study indicated that one or both parents of heart patients were addicts in 23.5% of the cases and among the control group healthy children was 2.29%, using χ^2^ at P = 0.001, which indicates a significant difference (Table 2). From heart patients 8.8% had addicted mothers only and 18.2% of the cases had addicted fathers and mothers both. This was 1.04% for control group children having only addicted mothers and 1.25 with both addicted fathers and mothers (Table 3).

**Table 2.** Distribution of Patients and Control Group of the Study for Gender Addiction of Parents

	Control	Case	Total
**Gender**			
Male	293	138	431
Female	187	87	274
**Parent’s addiction**			
Yes	11	61	72
No	469	164	633

**Table 3.** Distribution of Heart Disease Patients and Control Group Based on Having an Addicted Family Member

	Control	Case	Total
**Parents’ addiction**			
Mother	5	20	25
Father and mother	6	41	47
**Not addicted**	469	164	633
**Total**	480	225	705

Table 4 shows the relationship between heart disease and type of drug abuse with χ^2^ test and result of P = 0.0001 the relationship is statistically significant. Table 5 shows the distribution of the most common kinds of heart diseases based on the addition of family members.

**Table 4.** Distribution of Type of Addiction and Use in Mothers of Heart Patients[Table-fn fn3581]

Features	Control	Case
**Type of mothers’ addiction**		
Smoking opium	1	18
Eating opium	3	10
Eating and smoking opium	6	21
Heroin and opium	1	11
**No addiction**	469	165
**Total**	480	225

P value = 0.0001

**Table 5.** Distribution of the Most Common Kinds of Heart Diseases Based on the Addition of Family Members[Table-fn fn3582]

	Type of Father’s Addition, No.			Type of Mother’s Addiction, No.				Addiction of the Parents, No.		Addiction of the Family Members, No.	
**Type of Addiction**	Smoking and Eating	Eating Opium	Smoking Opium	Opium and Heroin	Smoking and Eating	Eating Opium	Smoking Opium	Father and Mother	Mother	No	Yes
**ASD**	3	0	0	2	4	0	0	3	3	13	6
**PDA**	3	2	4	0	4	2	4	9	1	28	10
**PS**	0	0	1	0	0	0	1	1	0	12	1
**TF**	1	0	2	0	2	0	2	3	1	23	4
**VSD**	7	8	4	7	10	8	6	19	12	67	31
**Total**	14	10	11	9	20	10	13	35	17	143	51

Abbreviations: ASD; atrial septal defect, PDA; patent ductus arteriosus, PS; pulmonary stenosis,TF; tetralogy of fallot, VSD; ventricular septal defect.

## 5. Discussion

From 225 children under the study 23.5% had addicted parents based on the variables of the study but the rate was only 2.3 for the control group. The difference between these two groups was significant. The number of children whose parents were both addicts, was more than those having only an addicted mother. Which was true about the patients and the control group as well. The most common form of drug abuse was smoking/eating opium and the most common form of heart disease was congenital ventricular septal defect (VSD). Opioids and most specifically morphine, the most effective component of opium, is hugely transferred through placenta to the fetus and is accumulated in the tissues of fetus especially in the heart (10). In the animal model studies, to examine the molecular mechanisms of drug-induced cardiac defects, the authors recently established a model in which cardiac defects were induced at a high incidence in rats following maternal administration of a compound. Embryonic exposure to dimethadione between gestational days (8 and 11) was found to induce a high incidence of ventricular septal defects (VSD) without significant maternal toxicity, and with minimal noncardiac fetal effects. VSD was the most common finding in response to dimethadione; however, many VSDs were together with by cardiac anomalies, such as outflow tract malformations, transposition of the great arteries, thinning of the myocardial wall, and right ventricular hypoplasia. This model mirrors the clinical manifestation of CHDs with reverence to the unity of VSDs and the spectrum of heart malformations observed. Moreover, it resembles the window during which dimethadione exposure causes a high incidence of heart and skeletal anomalies. The cardiac malformations included VSDs, conotruncal anomalies and small ventricles (9). In human studies akin animal researches showed that congenital cardiac malformation occurred in children who had addicted mothers. The current study showed that there was association between congenital cardiac anomalies and infants who had drug abuser mothers.

The teratogenic effect of opium and its derivates on cell proliferation in the first phase of pregnancy has been proven and suggests the possibility of similar effects on other systems in fetus (11). Among the most influenced body systems are the heart and vessles (11, 12). Vucinovic et al, revealed that children who had addicted mothers generally had lower birth weight with a seven-fold risk of small for gestational age newborns. The risk of numerous congenital anomalies was four-fold in the group of children who had addicted mothers. Congenital cardiac defects were most common, including ventriculoseptal defect, transposition of great vessels and hypoplastic left heart (13). Similar to which in the current study, there was correlation between congenital cardiac malformation and the children who had addicted mothers. Considering the effect of opioids and most especially opium on cell migration and proliferation in the first 20 weeks of pregnancy and also their effect on cell evolution and differentiation, opium’s teratogenicity effect on heart and vessels can be proved (3, 6, 7, 14, 15). Cardiovascular systems are created from the mesoderm germ layer in cephalic area of the fetus in the mid-week three and when the embryo can no longer satisfy its needs through propagation (15). After the formation of neural plate and subsequently formation of primary cerebral vesicle of central nervous system, the main heart tissues are formed. This system revolves 180 degrees along transverse axis and is pulled forward (12). This complicated process of cell division and proliferation suggests the possibility of teratogenicity effect of opium on various areas of the heart.

On the other hand, endocardial cushions cells originate from neural crest cells on which the effect of teratogenicity of opioids has been proven. This adds to the possibility of opium’s effect on heart and can explain the larger number of heart-wall defects in VSD. In addition, development of heart is initiated by various factors, the most important of which are polyamines necessary for heart and vessels remodeling including matrix development and heart tissues on which heart development relies (13, 14). Due to the Opioids and their transfer through mother’s body and by placenta, modification of OCD function occurs resulting in delay in heart’s development and reduction of its weight (16). Some previous investigations showed association between early pregnancy maternal opioid analgesic treatment and birth defect (13). 

The study of Thaithumyanon et al. showed that congenital abnormalities are found in amphetamine exposed infants. Up to the present time, no clear teratogenic effects of either heroin or amphetamine have been recognized. Similar to cocaine, amphetamine causes vasoconstriction via increasing circulating level of nor-epinephrine, serotonin and dopamine. If this effect happened during the period of organogenesis (18 - 54 days of gestation) of the embryo, abnormality could be noted. Malformations reported with the use of amphetamine during pregnancy contain cleft lip and palate, cardiac defects, biliary atresia and meningomyelocele (17). The findings of this study were, to about of, similar to our research in the children born of addicted mothers.

In another study Akin and Diehl - Jones showed that dissimilar screening possibilities exist for cocaine and its metabolites, including sampling of neonatal urine, hair and meconium need to be considered, as do the sensitivity and the ethical suggestions of such testing. Clinical management of cocaine-exposed infants requires consideration to several subjects, including: central nervous system irritation, cardiac anomalies, apnea, and feeding difficulties, as well as infant safety and follow-up postdischarge. Early detection and intervention remain the primary objectives of caring for cocaine-exposed infants (18). In the current study similar to that of Akin and Diehl - Jones, there was correlation between cardiac malformation and other symptoms in children who had addicted mothers. Another important point of this study was observing abnormalities in people whose mothers smoked and ate opium simultaneously indicating their addiction to higher doses of the drug during pregnancy and it can be also suggested that higher doses can cause congenital abnormalities, as well as damages to nervous system. 

The findings of the study showed that there is a statistically meaningful correlation between opium addition and congenital heart problems and also the amount of doses used. However, more comprehensive studies are required to clarify accurate molecular and genetic dimension of these problems like investigating animal models.
